# WHO publishes 3rd edition of *Trichiasis surgery for trachoma*

**Published:** 2024-10-02

**Authors:** Amir Bedri Kello, Shannath L Merbs, Lamine Traoré, Timothy Jesudason

**Affiliations:** 1 Medical Officer: Trachoma, World Health Organization Africa Regional Office, Brazzaville, Republic of Congo.; 2 Oculoplastic Surgeon: University of Maryland School of Medicine, Baltimore, USA.; 3 Programme National de la Santé Oculaire, Ministère de la Santé, Bamako, Mali.; 4 Special Projects and Campaign Partnerships: International Coalition for Trachoma Control, London, United Kingdom.

**Updates include the new definition of trachomatous trichiasis, and specific learning objectives and practical exercises for surgeons.**

**Figure:**
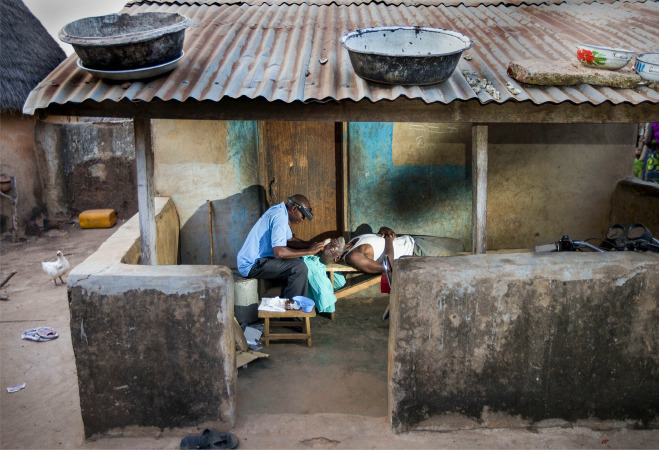
A trichiasis patient receives surgery outside his house. GHANA

© PETER NICHOLLS, SIGHTSAVERS CC BY-NC-SA 4.0

In May 2024, the World Health Organization published the third edition of *Trichiasis surgery for trachoma*. The manual incorporates recent evidence and developments in the field of trachomatous trichiasis (TT) surgery and provides detailed information for trainers and trainees on surgery for TT.^1^

The manual was first published in 1993, providing a detailed description of the bilamellar tarsal rotation (BLTR) procedure. In 2015, the manual was updated to include the TT and Waddell clamps for the BLTR procedure, as well as adding the posterior lamellar tarsal rotation (PLTR) or modified Trabut procedure. Additionally, to improve the quality of TT surgery conducted globally, a final assessment of TT surgeons was included.

The third edition of *Trichiasis surgery for trachoma* – also commonly known as the ‘Yellow Manual’ – is divided into two parts. Part One details the recommended surgical techniques, BLTR and PLTR, and covers the specific skills that TT surgeons should possess in order to be certified. Guiding both trainers and trainees, the manual provides specific learning objectives and practical exercises, as well as describing the skills that should be developed and assessed. Part Two was developed specifically for trainers and includes information about the selection and final assessments of trainees.

During the fourth Global Scientific Meeting on Trachoma, held in Geneva in 2018, global stakeholders agreed to a new definition of TT: at least one eyelash from the upper eyelid touches the eyeball or there is evidence of recent epilation of in-turned eyelashes from the upper eyelid.

**“Another new addition to the manual is the recommendation that follow-up on the first day after surgery is done by the surgeon who performed the operation.”**

In addition to including this new definition, the revised manual now defines entropion as the condition when part or all of the eyelid margin is turned inwards and not visible and describes how to examine for entropion. Because not all eyes with TT also have entropion, the identification of entropion is important, since the main purpose of TT surgery is to correct trichiasis and entropion by making an incision and rotating the eyelid margin outward. Therefore, the presence of entropion is now included as an indicator for TT surgery.

The updated indicators for TT surgery in the community are when:

- one or more eyelashes from the upper eyelid turn in and touch the cornea when the patient looks straight ahead, plus evidence of entropion
- there is evidence of corneal damage from TT, or
- the patient has severe discomfort from TT.

Another new addition to the manual is the recommendation that follow-up on the first day after surgery is done by the surgeon who performed the operation. To ensure high-quality outcomes, the manual includes guidance on recognising and correcting both undercorrection and overcorrection at this first follow-up visit, as well as guidance on how to identify later complications, such as postoperative TT and surgical complications following TT surgery.

The global neglected tropical disease (NTD) road map sets a target of eliminating trachoma as a public health problem by 2030. This includes reducing the prevalence of TT in people aged >15 years to less than 0.2% and providing evidence that health systems can continue to identify and manage incident cases of TT. As countries continue to accelerate towards these goals, the third edition of *Trichiasis surgery for trachoma* will support national programmes in the training of TT surgeons to conduct high quality surgery.
